# Supplementary material for: Fractal dimension analysis of different mandibular regions in familial Mediterranean fever patients: A cross-sectional retrospective study
Source: PLoS One. 2023 Jun 30;18(6):e0288170. doi: 10.1371/journal.pone.0288170 (PMC10313079; doi:10.1371/journal.pone.0288170)
Supplement: S1 Checklist — (DOCX) [file pone.0288170.s001.docx]

STROBE Statement—checklist of items that should be included in reports of observational studies

|  | **Item No.** | **Recommendation** | **Page  No.** | **Relevant text from manuscript** |
| --- | --- | --- | --- | --- |
| **Title and abstract** | 1 | (*a*) Indicate the study’s design with a commonly used term in the title or the abstract | 1,2 | (Line# 4,5,24-27)  Fractal dimension analysis of different mandibular regions in familial Mediterranean fever patients: A cross-sectional retrospective study  The purpose of this cross-sectional retrospective study was to evaluate trabecular and cortical microarchitecture of the mandible with FD analysis on panoramic radiographs in a subpopulation of FMF. |
|  |  | (*b*) Provide in the abstract an informative and balanced summary of what was done and what was found | 2 | (Line# 27-39)  Forty-three FMF patients, aged between 10.8 and 71.2 years, and age- and gender-matched control group consisting of patients, who had no systemic diseases, were included. Demographic information such as age and gender, and colchicine use were recorded. In terms of age, the patients were classified as <30 and 30< years. On each panoramic radiographs five regions of interest were selected on the mandible as: 1- premolar, 2- molar, 3- angular, 4- condylar, and 5- basal cortical bone regions on right (R) and left (L) sides. Statistical significance was accepted at p<0.05 level. Intra- and inter-observer agreements demonstrated good to excellent consistency. In FMF patients, L3 and L4 values were higher, whereas L5 values were lower (p<0.05) than the control group. In terms of age, the difference between groups was insignificant in FMF patients (p>0.05), whereas in control group R3 and L4 values were higher in the 30< age group (p<0.05). Regarding gender and colchicine use, the difference between groups was insignificant (p>0.05). |
| **Introduction** | | | |  |
| Background/rationale | 2 | Explain the scientific background and rationale for the investigation being reported | 3-5 | (Line# 63-102)  Chronic subclinical inflammation in FMF patients might also give rise to decreased bone mineral density (BMD) and osteoporosis by affecting bone turnover and metabolism [10,11]. Many authors showed low levels of BMD and bone formation markers in individuals with FMF [11-13]. Colchicine decreases osteoclast numbers and inhibits resorption in bones [14,15], and therefore improve bone density and prevent osteoporosis [4,11,13].  Diagnosis of osteoporosis at an early stage is an important healthcare issue. The current principal method for diagnosing osteoporosis is measurement of BMD by dual energy X-ray absorptiometry (DXA) [16]. DXA is the gold standard for BMD measurements and is used for the diagnosis of osteopenia and osteoporosis, as well as prediction the fracture risk. The distribution of the equipment and cost of advanced imaging techniques, such as DXA, limit their access for screening of larger populations [17].  Another method to determine the changes related to bone mineral loss is fractal dimension (FD) analysis on panoramic radiographs. Panoramic radiography is one of the most used imaging modalities in dentistry and is an important part of routine dental care. It provides a valuable screening opportunity due to its lower cost and radiation dose, as well as a high access. Fractal analysis, which is a mathematical image analysis method, is used in the analysis of complex shapes and structural formations. The interpretation of the mandible on a panoramic image via computational analysis methods gives objective numerical results defined as the FD, and thus rules out the subjective judgment of the observer [18]. FD can assist in the quantification of complex structures and description of bone microarchitecture and demonstrate bone mineral loss on panoramic images [19]. And thus, FD is considered to be a distinctive parameter for the determination of the density of the osteoporotic and normal bone tissue [20,21]. The increased FD values have been linked to the increased complexity of the structure. A recent systematic review concluded that FD analysis on dental images is a reliable diagnostic tool for osteoporosis screening and could be a reference BMD test [22]. Even though both trabecular and cortical osseous tissue have a fractal structure, this structure is not visible to the naked eye for cortical bone [23]. In previous studies mostly trabecular bone regions were selected for the FD evaluation, while there are also studies including the cortical bone areas, as well [20,24-32]. Sindeaux et al. [30] demonstrated that FD values of the cortical bone were more accurate than those of the trabecular bone. They also reported that the patients with osteoporosis have higher probability of having lower mean FD values on the cortical bone compared to healthy counterparts and that cortical bone FD measurements might be considered as auxiliary tools to refer patients for DXA exam [30].  To the best of our knowledge, there is only one study that evaluates the bone microarchitecture of FMF patients using FD analysis that was performed on the trabecular structure of the mandible via panoramic radiographs of solely pediatric patients [33]. To date, no study exists, in which FD analysis was applied on panoramic radiographs of adult FMF patients, taking the colchicine use and mandibular cortical structure into consideration, as well. |
| Objectives | 3 | State specific objectives, including any prespecified hypotheses | 4 | (Line# 102-107)  The hypothesis was that there were no differences in the microarchitecture of the mandible between FMF and healthy populations. Therefore, the aim of this cross-sectional retrospective study was to assess trabecular and cortical bone structures on panoramic radiographs in a subpopulation of FMF in order to find out whether significant differences exist in FMF patients and healthy individuals. Also, the effect of colchicine use on mandibular cortical and trabecular bone microarchitecture was investigated in FMF patients. |
| **Methods** | | | |  |
| Study design | 4 | Present key elements of study design early in the paper | 5-8 | (Line# 117-141,145-183)  Sample size calculation performed by G* Power 3.1.9.2 (Kiel, Germany) revealed that a total of minimum 50 panoramic radiographs from FMF and control patients (n=25 in each group) was necessary (confidence interval: 95%, significance level: 0.05, effect size (d): 0.47).  The database of the Department of Dentomaxillofacial Radiology of the Yeditepe University Faculty of Dentistry was retrospectively reviewed in August-September, 2022. Overall 75 self-reported FMF patients, who underwent panoramic imaging at Yeditepe University Faculty of Dentistry between January 2014 and November 2019 and have a panoramic radiograph with a good image quality, were identified. Among these patients, 32 of them, who had systemic diseases that affect bone metabolism (15 patients), and were using medications affecting the bone metabolism (3 patients), as well as the patients with a mixed dentition (13 patients) and severely atrophic alveolar crest (1 patient) that complicated the measurements on panoramic radiographs, were excluded. As a result, 43 FMF patients aged between 10-71 years and a control group consisting of the same number of randomly selected age-gender-matched patients, who had no systemic diseases, were included in the study. Control group, which was also subjected to the exclusion criteria, was selected randomly from the patients in the same database, depending on the matching characteristics of the patients in the FMF group, in terms of age and gender by filtering the data on Microsoft Excel sheet (Microsoft Office Professional Plus 2010 v14.0, Microsoft Corporation). The remaining patients were assigned a number consequently and control patients corresponding to the FMF patients were randomly selected by using a random number generator (random.org). Demographic information such as age and gender, and colchicine use were recorded. The patients were further classified into two age groups as <30 and 30< years. Besides, the patients with FMF were classified according to colchicine use. Self-reported colchicine use was recorded as ‘Yes’ or ‘No’, without taking the prior use or dose regimen into consideration.  A total of 86 panoramic radiographic images obtained with Planmeca 2002 cc Proline (Planmeca, Helsinki, Finland; 70 kVp, 10 mA, 8 s exposure time) and x1.34 magnification factor, were evaluated. Orientation of the head was arranged so that the Frankfort horizontal plane was parallel to the floor and the sagittal plane was parallel to the vertical plane. Digital images were exported in 8-bit depth grayscale high resolution ‘.tiff’ format from the Planmeca Romexis 3.8.3 (Helsinki, Finland).  The FD measurements were performed on the ImageJ software (ImageJ 1.38; US National Institutes of Health, Bethesda, MD, USA) that was downloaded from https://imagej.nih.gov/ij/download.html. All sets of the fully anonymized panoramic images were imported into ImageJ software. Four different regions of interest (ROI) in 30x30 pixel size were selected from designated spots (rectangle tool) on right (R) and left (L) side of the mandible as follows (Fig 2); ROI1: Distal region of the premolar, next to the mental foramen, ROI2: mesial region of the apical part of second molar, ROI3: angular region of the mandible, and ROI4: mandibular condyle area. An additional fifth ROI was selected in differing pixel size depending on the ROI (polygon tool) on each side of the mandible as; ROI5: basal cortical bone of the mandible extending from distal to the mental foramen to the distal root of the first molar (Fig 2). The individual in this manuscript has given written informed consent to publish these case details. Each ROI was selected and the FD analysis was conducted as follows; duplication of the selected ROI, application of the Gaussian filter [34] to remove brightness alterations due to overlying soft and hard tissue, subtraction of the filtered image from the original cropped image, addition of a gray value of 128 to differentiate bone marrow spaces and trabeculae, binarization of the resulting image, steps of erosion, dilatation, inversion, and skeletonization. Lastly, the FD was calculated according to the fractal box counting method described by White & Rudolph [34]. The FD analysis was carried out by two independent observers (a dentomaxillofacial radiology specialist with 12-year of experience and an endodontist with an 8-year of experience). Prior to the FD analysis, the observers were calibrated by evaluating 15 panoramic radiographs, which were not included in the study, together. After the first independent readings 25% of the measurements were repeated after a two-week interval for intra- and inter-observer repeatability. Data collection was completed in November, 2022. FD analysis on panoramic radiographs was comparable for both FMF and control groups that all the fully anonymized panoramic images were evaluated in the same manner by two observers. The observers had access to patient information that could identify individual participants after data collection.  The fact that the information regarding the diagnosis of FMF and colchicine use, as well as other possible diseases and drug use questioned during taking anamnesis and evaluated in the exclusion criteria was based on the self-reported information was a potential confounder and a limitation of the study. Age was another confounder that we tried to overcome by selecting age and gender matched patients in the control group. Also, we classified the patients in two age groups as <30 and 30< years. |
| Setting | 5 | Describe the setting, locations, and relevant dates, including periods of recruitment, exposure, follow-up, and data collection | 6-8 | (Line 121-125,131-137,145-153,173-174,185)  The database of the Department of Dentomaxillofacial Radiology of the Yeditepe University Faculty of Dentistry was retrospectively reviewed in August-September, 2022. Overall 75 self-reported FMF patients, who underwent panoramic imaging at Yeditepe University Faculty of Dentistry between January 2014 and November 2019 and have a panoramic radiograph with a good image quality, were identified.  Control group, which was also subjected to the exclusion criteria, was selected randomly from the patients in the same database, depending on the matching characteristics of the patients in the FMF group, in terms of age and gender by filtering the data on Microsoft Excel sheet (Microsoft Office Professional Plus 2010 v14.0, Microsoft Corporation). The remaining patients were assigned a number consequently and control patients corresponding to the FMF patients were randomly selected by using a random number generator (random.org).  A total of 86 panoramic radiographic images obtained with Planmeca 2002 cc Proline (Planmeca, Helsinki, Finland; 70 kVp, 10 mA, 8 s exposure time) and x1.34 magnification factor, were evaluated. Orientation of the head was arranged so that the Frankfort horizontal plane was parallel to the floor and the sagittal plane was parallel to the vertical plane. Digital images were exported in 8-bit depth grayscale high resolution ‘.tiff’ format from the Planmeca Romexis 3.8.3 (Helsinki, Finland).  The FD measurements were performed on the ImageJ software (ImageJ 1.38; US National Institutes of Health, Bethesda, MD, USA) that was downloaded from https://imagej.nih.gov/ij/download.html.  Data collection was completed in November, 2022.  SPSS software version 25.0 (IBM, USA) was used for the statistical analysis. |
| Participants | 6 | (*a*) *Cross-sectional study*—Give the eligibility criteria, and the sources and methods of selection of participants | 6 | (Line# 121-137)  The database of the Department of Dentomaxillofacial Radiology of the Yeditepe University Faculty of Dentistry was retrospectively reviewed in August-September, 2022. Overall 75 self-reported FMF patients, who underwent panoramic imaging at Yeditepe University Faculty of Dentistry between January 2014 and November 2019 and have a panoramic radiograph with a good image quality, were identified. Among these patients, 32 of them, who had systemic diseases that affect bone metabolism (15 patients), and were using medications affecting the bone metabolism (3 patients), as well as the patients with a mixed dentition (13 patients) and severely atrophic alveolar crest (1 patient) that complicated the measurements on panoramic radiographs, were excluded. As a result, 43 FMF patients aged between 10-71 years and a control group consisting of the same number of randomly selected age-gender-matched patients, who had no systemic diseases, were included in the study. Control group, which was also subjected to the exclusion criteria, was selected randomly from the patients in the same database, depending on the matching characteristics of the patients in the FMF group, in terms of age and gender by filtering the data on Microsoft Excel sheet (Microsoft Office Professional Plus 2010 v14.0, Microsoft Corporation). The remaining patients were assigned a number consequently and control patients corresponding to the FMF patients were randomly selected by using a random number generator (random.org). |
| Variables | 7 | Clearly define all outcomes, exposures, predictors, potential confounders, and effect modifiers. Give diagnostic criteria, if applicable | 6-8 | (Line# 122-141,148-151,153-161,168-183)  Overall 75 self-reported FMF patients, who underwent panoramic imaging at Yeditepe University Faculty of Dentistry between January 2014 and November 2019 and have a panoramic radiograph with a good image quality, were identified. Among these patients, 32 of them, who had systemic diseases that affect bone metabolism (15 patients), and were using medications affecting the bone metabolism (3 patients), as well as the patients with a mixed dentition (13 patients) and severely atrophic alveolar crest (1 patient) that complicated the measurements on panoramic radiographs, were excluded. As a result, 43 FMF patients aged between 10-71 years and a control group consisting of the same number of randomly selected age-gender-matched patients, who had no systemic diseases, were included in the study. Control group, which was also subjected to the exclusion criteria, was selected randomly from the patients in the same database, depending on the matching characteristics of the patients in the FMF group, in terms of age and gender by filtering the data on Microsoft Excel sheet (Microsoft Office Professional Plus 2010 v14.0, Microsoft Corporation). The remaining patients were assigned a number consequently and control patients corresponding to the FMF patients were randomly selected by using a random number generator (random.org). Demographic information such as age and gender, and colchicine use were recorded. The patients were further classified into two age groups as <30 and 30< years. Besides, the patients with FMF were classified according to colchicine use. Self-reported colchicine use was recorded as ‘Yes’ or ‘No’, without taking the prior use or dose regimen into consideration.  Digital images were exported in 8-bit depth grayscale high resolution ‘.tiff’ format from the Planmeca Romexis 3.8.3 (Helsinki, Finland).  The FD measurements were performed on the ImageJ software  All sets of the fully anonymized panoramic images were imported into ImageJ software. Four different regions of interest (ROI) in 30x30 pixel size were selected from designated spots (rectangle tool) on right (R) and left (L) side of the mandible as follows (Fig 2); ROI1: Distal region of the premolar, next to the mental foramen, ROI2: mesial region of the apical part of second molar, ROI3: angular region of the mandible, and ROI4: mandibular condyle area. An additional fifth ROI was selected in differing pixel size depending on the ROI (polygon tool) on each side of the mandible as; ROI5: basal cortical bone of the mandible extending from distal to the mental foramen to the distal root of the first molar (Fig 2).  The FD analysis was carried out by two independent observers (a dentomaxillofacial radiology specialist with 12-year of experience and an endodontist with an 8-year of experience). Prior to the FD analysis, the observers were calibrated by evaluating 15 panoramic radiographs, which were not included in the study, together. After the first independent readings 25% of the measurements were repeated after a two-week interval for intra- and inter-observer repeatability. Data collection was completed in November, 2022. FD analysis on panoramic radiographs was comparable for both FMF and control groups that all the fully anonymized panoramic images were evaluated in the same manner by two observers. The observers had access to patient information that could identify individual participants after data collection.  The fact that the information regarding the diagnosis of FMF and colchicine use, as well as other possible diseases and drug use questioned during taking anamnesis and evaluated in the exclusion criteria was based on the self-reported information was a potential confounder and a limitation of the study. Age was another confounder that we tried to overcome by selecting age and gender matched patients in the control group. Also, we classified the patients in two age groups as <30 and 30< years. |
| Data sources/ measurement | 8* | For each variable of interest, give sources of data and details of methods of assessment (measurement). Describe comparability of assessment methods if there is more than one group | 6-8 | (Line# 121-125,129-131,148-177)  The database of the Department of Dentomaxillofacial Radiology of the Yeditepe University Faculty of Dentistry was retrospectively reviewed in August-September, 2022. Overall 75 self-reported FMF patients, who underwent panoramic imaging at Yeditepe University Faculty of Dentistry between January 2014 and November 2019 and have a panoramic radiograph with a good image quality, were identified. Among these patients, 32 of them were excluded. As a result, 43 FMF patients aged between 10-71 years and a control group consisting of the same number of randomly selected age-gender-matched patients, who had no systemic diseases, were included in the study.  Digital images were exported in 8-bit depth high resolution ‘.tiff’ format from the Planmeca Romexis 3.8.3 (Helsinki, Finland).  The FD measurements were performed on the ImageJ software (ImageJ 1.38; US National Institutes of Health, Bethesda, MD, USA) that was downloaded from https://imagej.nih.gov/ij/download.html. All sets of the fully anonymized panoramic images were imported into ImageJ software. Four different regions of interest (ROI) in 30x30 pixel size were selected from designated spots (rectangle tool) on right (R) and left (L) side of the mandible as follows (Fig 2); ROI1: Distal region of the premolar, next to the mental foramen, ROI2: mesial region of the apical part of second molar, ROI3: angular region of the mandible, and ROI4: mandibular condyle area. An additional fifth ROI was selected in differing pixel size depending on the ROI (polygon tool) on each side of the mandible as; ROI5: basal cortical bone of the mandible extending from distal to the mental foramen to the distal root of the first molar (Fig 2). The individual in this manuscript has given written informed consent to publish these case details. Each ROI was selected and the FD analysis was conducted as follows; duplication of the selected ROI, application of the Gaussian filter [34] to remove brightness alterations due to overlying soft and hard tissue, subtraction of the filtered image from the original cropped image, addition of a gray value of 128 to differentiate bone marrow spaces and trabeculae, binarization of the resulting image, steps of erosion, dilatation, inversion, and skeletonization. Lastly, the FD was calculated according to the fractal box counting method described by White & Rudolph [34]. The FD analysis was carried out by two independent observers (a dentomaxillofacial radiology specialist with 12-year of experience and an endodontist with an 8-year of experience). Prior to the FD analysis, the observers were calibrated by evaluating 15 panoramic radiographs, which were not included in the study, together. After the first independent readings 25% of the measurements were repeated after a two-week interval for intra- and inter-observer repeatability. Data collection was completed in November, 2022. FD analysis on panoramic radiographs was comparable for both FMF and control groups that all the fully anonymized panoramic images were evaluated in the same manner by two observers. The observers had access to patient information that could identify individual participants after data collection. |
| Bias | 9 | Describe any efforts to address potential sources of bias | 6-8 | Line# 122-137,168-183)  All the FMF patients, who underwent panoramic imaging at Yeditepe University Faculty of Dentistry between January 2014 and November 2019, have a panoramic radiograph with a good image quality, and do not meet the exclusion criteria, were included.  Control group, which was also subjected to the exclusion criteria, was selected randomly from the patients in the same database, depending on the matching characteristics of the patients in the FMF group, in terms of age and gender by filtering the data on Microsoft Excel sheet (Microsoft Office Professional Plus 2010 v14.0, Microsoft Corporation). The remaining patients were assigned a number consequently and control patients corresponding to the FMF patients were randomly selected by using a random number generator (random.org).  The FD analysis was carried out by two independent observers (a dentomaxillofacial radiology specialist with 12-year of experience and an endodontist with an 8-year of experience). Prior to the FD analysis, the observers were calibrated by evaluating 15 panoramic radiographs, which were not included in the study, together. After the first independent readings 25% of the measurements were repeated after a two-week interval for intra- and inter-observer repeatability. Data collection was completed in November, 2022. FD analysis on panoramic radiographs was comparable for both FMF and control groups that all the fully anonymized panoramic images were evaluated in the same manner by two observers. The observers had access to patient information that could identify individual participants after data collection.  The fact that the information regarding the diagnosis of FMF and colchicine use, as well as other possible diseases and drug use questioned during taking anamnesis and evaluated in the exclusion criteria was based on the self-reported information was a potential confounder and a limitation of the study. Age was another confounder that we tried to overcome by selecting age and gender matched patients in the control group. Also, we classified the patients in two age groups as <30 and 30< years. (Unfortunately the bias caused by the determination of the FMF diagnosis and the prior use of colchicine and colchicine use regimen could not be addressed) |
| Study size | 10 | Explain how the study size was arrived at | 5,6 | (Line# 117-131)  Sample size calculation performed by G* Power 3.1.9.2 (Kiel, Germany) revealed that a total of minimum 50 panoramic radiographs from FMF and control patients (n=25 in each group) was necessary (confidence interval: 95%, significance level: 0.05, effect size (d): 0.47).  The database of the Department of Dentomaxillofacial Radiology of the Yeditepe University Faculty of Dentistry was retrospectively reviewed in August-September, 2022. Overall 75 self-reported FMF patients, who underwent panoramic imaging at Yeditepe University Faculty of Dentistry between January 2014 and November 2019 and have a panoramic radiograph with a good image quality, were identified. Among these patients, 32 of them, who had systemic diseases that affect bone metabolism (15 patients), and were using medications affecting the bone metabolism (3 patients), as well as the patients with a mixed dentition (13 patients) and severely atrophic alveolar crest (1 patient) that complicated the measurements on panoramic radiographs, were excluded. As a result, 43 FMF patients aged between 10-71 years and a control group consisting of the same number of randomly selected age-gender-matched patients, who had no systemic diseases, were included in the study. |

Continued on next page

| Quantitative variables | 11 | | Explain how quantitative variables were handled in the analyses. If applicable, describe which groupings were chosen and why | 6-8 | (Line# 129-131,137-141,145-174)  43 FMF patients aged between 10-71 years and a control group consisting of the same number of randomly selected age-gender-matched patients, who had no systemic diseases, were included in the study.  Demographic information such as age and gender, and colchicine use were recorded. The patients were further classified into two age groups as <30 and 30< years. Besides, the patients with FMF were classified according to colchicine use. Self-reported colchicine use was recorded as ‘Yes’ or ‘No’, without taking the prior use or dose regimen into consideration. (The patients were further classified according to gender as female and male, and into two age groups as <30 and 30< years in order to find out whether differences exist in terms of gender or age groups, as well as to address the potential sources of bias. Besides, the patients with FMF were classified according to colchicine use, which might also have an effect on the bone tissue, therefore on the measurements.)  A total of 86 panoramic radiographic images were evaluated. Digital images were exported in 8-bit depth high resolution ‘.tiff’ format from the Planmeca Romexis 3.8.3 (Helsinki, Finland).  The FD measurements were performed on the ImageJ software (ImageJ 1.38; US National Institutes of Health, Bethesda, MD, USA) that was downloaded from https://imagej.nih.gov/ij/download.html. All sets of the fully anonymized panoramic images were imported into ImageJ software. Four different regions of interest (ROI) in 30x30 pixel size were selected from designated spots (rectangle tool) on right (R) and left (L) side of the mandible as follows [16] (Fig 2); ROI1: Distal region of the premolar, next to the mental foramen, ROI2: mesial region of the apical part of second molar, ROI3: angular region of the mandible, and ROI4: mandibular condyle area. An additional fifth ROI was selected in differing pixel size depending on the ROI (polygon tool) on each side of the mandible as; ROI5: basal cortical bone of the mandible extending from distal to the mental foramen to the distal root of the first molar (Fig 2). Each ROI was selected and the FD analysis was conducted as follows; duplication of the selected ROI, application of the Gaussian filter [24] to remove brightness alterations due to overlying soft and hard tissue, subtraction of the filtered image from the original cropped image, addition of a gray value of 128 to differentiate bone marrow spaces and trabeculae, binarization of the resulting image, steps of erosion, dilatation, inversion, and skeletonization. Lastly, the FD was calculated according to the fractal box counting method described by White & Rudolph [20]. The FD analysis was carried out by two independent observers (a dentomaxillofacial radiology specialist with 12-year of experience and an endodontist with an 8-year of experience). Prior to the FD analysis, the observers were calibrated by evaluating 15 panoramic radiographs, which were not included in the study, together. After the first independent readings 25% of the measurements were repeated after a two-week interval for intra- and inter-observer repeatability. Data collection was completed in November, 2022. |
| --- | --- | --- | --- | --- | --- |
| Statistical methods | 12 | | (*a*) Describe all statistical methods, including those used to control for confounding | 5,6,8 | (Line# 100-103,185-193)  Sample size calculation performed by G* Power 3.1.9.2 (Kiel, Germany) revealed that a total of minimum 50 panoramic radiographs from FMF and control patients (n=25 in each group) was necessary (confidence interval: 95%, significance level: 0.05, effect size (d): 0.47).  SPSS software version 25.0 (IBM, USA) was used for the statistical analysis. Normality distribution of all variables was analyzed with histogram graphics and Kolmogorov-Smirnov tests. Mean, standard deviation, median, and IQR values were used for descriptive analysis. Analysis of the nonparametric variables that did not display a normal distribution among two groups was performed with Mann-Whitney U test, whereas Independent t-test was performed to analyze parametric variables that were normally distributed. Chi-square test was used to assess the distribution of categorical data between FMF and control groups. Intra- and inter-observer agreement was assessed with Intraclass Correlation Coefficient (ICC) [35]. Statistical significance was accepted at p<0.05 level. |
|  |  |  | (*b*) Describe any methods used to examine subgroups and interactions | 8 | (Line# 188-193)  Analysis of the nonparametric variables that did not display a normal distribution among two groups was performed with Mann-Whitney U test, whereas Independent t-test was performed to analyze parametric variables that were normally distributed. Chi-square test was used to assess the distribution of categorical data between FMF and control groups. Intra- and inter-observer agreement was assessed with Intraclass Correlation Coefficient (ICC) [35]. Statistical significance was accepted at p<0.05 level. |
|  |  |  | (*c*) Explain how missing data were addressed | - | No missing data eligible to be addressed. |
|  |  |  | (*d*) *Cross-sectional study*—If applicable, describe analytical methods taking account of sampling strategy | 5,6 | (Line# 117-120)  Sample size calculation performed by G* Power 3.1.9.2 (Kiel, Germany) revealed that a total of minimum 50 panoramic radiographs from FMF and control patients (n=25) was necessary (confidence interval: 95%, significance level: 0.05, effect size (d): 0.47). |
|  |  |  | (*e*) Describe any sensitivity analyses | 8 | (Line# 191-193)  Intra- and inter-observer agreement was assessed with Intraclass Correlation Coefficient (ICC) [25]. Statistical significance was accepted at p<0.05 level. |
| **Results** | | | | | |
| Participants | 13* | (a) Report numbers of individuals at each stage of study—eg numbers potentially eligible, examined for eligibility, confirmed eligible, included in the study, completing follow-up, and analysed | | 6,7,9 | (Line# 122-131,141-147,195-197)  Overall 75 self-reported FMF patients, who underwent panoramic imaging at Yeditepe University Faculty of Dentistry between January 2014 and November 2019 and have a panoramic radiograph with a good image quality, were identified. Among these patients, 32 of them, who had systemic diseases that affect bone metabolism (15 patients), and were using medications affecting the bone metabolism (3 patients), as well as the patients with a mixed dentition (13 patients) and severely atrophic alveolar crest (1 patient) that complicated the measurements on panoramic radiographs, were excluded. As a result, 43 FMF patients aged between 10-71 years and a control group consisting of the same number of randomly selected age-gender-matched patients, who had no systemic diseases, were included in the study.  A flow chart of the study design demonstrating the eligibility and the numbers of individuals recruited at each stage of study (Fig 1).  Figure 1. Flow chart of the study design.  A total of 86 panoramic radiographic images were evaluated.  Demographic distribution of the patients is demonstrated on Table 1.  Table 1. Demographic distribution of the FMF and control patients. |
|  |  | (b) Give reasons for non-participation at each stage | | 6 | (Line# 125-129)  Among these patients, 32 of them, who had systemic diseases that affect bone metabolism (15 patients), and were using medications affecting the bone metabolism (3 patients), as well as the patients with a mixed dentition (13 patients) and severely atrophic alveolar crest (1 patient) that complicated the measurements on panoramic radiographs, were excluded |
|  |  | (c) Consider use of a flow diagram | | 6,7 | (Line# 141-144)  A flow chart of the study design demonstrating the eligibility and the numbers of individuals recruited at each stage of study (Fig 1).  Figure 1. Flow chart of the study design. |
| Descriptive data | 14* | (a) Give characteristics of study participants (eg demographic, clinical, social) and information on exposures and potential confounders | | 9 | (Line# 176-178)  Demographic distribution of the patients is demonstrated on Table 1.  Table 1. Demographic distribution of the FMF and control patients. |
|  |  | (b) Indicate number of participants with missing data for each variable of interest | | - | None. |
| Outcome data | 15* | *Cross-sectional study—*Report numbers of outcome events or summary measures | | 9 | (Line# 204-206,212-214)  Comparison of the FMF and control groups regarding right and left ROI measurements revealed that in FMF patients mean L3 and L4 values were found to be higher, whereas mean L5 values were less than the control group (p<0.05, Table 3).  Table 3. Comparison of ROI measurements obtained on right and left sides on the panoramic radiographs of FMF and control groups. |
| Main results | 16 | (*a*) Give unadjusted estimates and, if applicable, confounder-adjusted estimates and their precision (eg, 95% confidence interval). Make clear which confounders were adjusted for and why they were included | | 8 | (Line# 178-183)  The fact that the information regarding the diagnosis of FMF and colchicine use, as well as other possible diseases and drug use questioned during taking anamnesis and evaluated in the exclusion criteria was based on the self-reported information was a potential confounder and a limitation of the study. Age was another confounder that we tried to overcome by selecting age and gender matched patients in the control group. Also, we classified the patients in two age groups as <30 and 30< years. (The patients were further classified according to gender as female and male, and into two age groups as <30 and 30< years in order to find out whether differences exist in terms of gender or age groups, as well as to address the potential sources of bias. Besides, the patients with FMF were classified according to colchicine use, which might also have an effect on the bone tissue, therefore on the measurements.)  No further adjustments were made on this issue. |
|  |  | (*b*) Report category boundaries when continuous variables were categorised | | 8 | (Line# 182-183)  We classified the patients in two age groups as <30 and 30< years. |
|  |  | (*c*) If relevant, consider translating estimates of relative risk into absolute risk for a meaningful time period | | - | Irrelevant. |

Continued on next page

| Other analyses | 17 | | Report other analyses done—eg analyses of subgroups and interactions, and sensitivity analyses | | 9-11 | (Line# 198-222)  Regarding all the ROI measurements, intra-observer agreement of the first and second observer, and inter-observer agreement that were determined using ICC test demonstrated a good to excellent agreement that ranged between 0.752-0.944, 0.802-0.958, and 0.765-0.962, respectively (p<0.05, Table 2).  Comparison of the FMF and control groups regarding right and left ROI measurements revealed that in FMF patients mean L3 and L4 values were found to be higher, whereas mean L5 values were less than the control group (p<0.05, Table 3). In terms of age groups, the difference between ROI measurements on both sides in FMF group was not significant (p>0.05), whereas in control group R3 and L4 values were found to be higher in 30< age group comparing to <30 age group (p<0.05, Table 4). In terms of gender in both groups and colchicine use in the FMF group, the difference between ROI measurements on right and left sides was insignificant (p>0.05, Tables 5 and 6).  Table 2. Intra- and inter-observer agreements determined using ICC test.  Table 3. Comparison of fractal dimension measurements obtained on right and left sides on the panoramic radiographs of FMF and control groups.  Table 4. Fractal dimension measurements in the FMF and control groups according to different age groups.  Table 5. Fractal dimension measurements in the FMF and control groups according to gender.  Table 6. Fractal dimension measurements in the FMF group according to colchicine use. | | | |
| --- | --- | --- | --- | --- | --- | --- | --- | --- | --- |
| **Discussion** | | | | | | | | | |
| Key results | 18 | Summarise key results with reference to study objectives | | 5,9-14 | | | | (Line# 102-107,204-222,249-252,272-275,277-278)  The hypothesis was that there were no differences in the microarchitecture of the mandible between FMF and healthy populations. Therefore, the aim of this cross-sectional retrospective study was to assess trabecular and cortical bone structures on panoramic radiographs in a subpopulation of FMF in order to find out whether significant differences exist in FMF patients and healthy individuals. Also, the effect of colchicine use on mandibular cortical and trabecular bone microarchitecture was investigated in FMF patients.  Comparison of the FMF and control groups regarding right and left ROI measurements revealed that in FMF patients mean L3 and L4 values were found to be higher, whereas mean L5 values were less than the control group (p<0.05, Table 3). In terms of age groups, the difference between ROI measurements on both sides in FMF group was not significant (p>0.05), whereas in control group R3 and L4 values were found to be higher in 30< age group comparing to <30 age group (p<0.05, Table 4). In terms of gender in both groups and colchicine use in the FMF group, the difference between ROI measurements on right and left sides was insignificant (p>0.05, Tables 5 and 6).  Table 3. Comparison of fractal dimension measurements obtained on right and left sides on the panoramic radiographs of FMF and control groups.  Table 4. Fractal dimension measurements in the FMF and control groups according to different age groups.  Table 5. Fractal dimension measurements in the FMF and control groups according to gender.  Table 6. Fractal dimension measurements in the FMF group according to colchicine use.  A significant difference was not observed in FMF patients, in terms of age groups or colchicine use. On the other hand, in terms of age, control patients revealed a greater R3 and L4 values in the patients aged 30< years.  In our study, in FMF patients similar FD values were demonstrated in terms of age, gender, and colchicine use. However, while in the left angulus and condylar area FD measurements were significantly greater, the left mandibular cortical area revealed lower FD measurements in the FMF patients.  In our study, L5 value, which concerns the mandibular cortical bone, was lower in the FMF patients. | |
| Limitations | 19 | Discuss limitations of the study, taking into account sources of potential bias or imprecision. Discuss both direction and magnitude of any potential bias | | 15 | | | | (Line# 302-310)  The limitation of the study was that due to the retrospective cross-sectional nature of the study the gathered data depended on self-reported medical records obtained during questioning of the systemic anamnesis at a dental hospital and detailed information regarding FMF diagnosis, laboratory or imaging results of the test that are more accurately define BMD or treatment dosage and regimen, or previous colchicine use could not be obtained and evaluated. Additionally, information related to dental anamnesis such as dental status, parafunctional habits, and chewing side preference of the patients, as well as clinical findings which would affect the morphology of the bone also were not recorded and evaluated in this study. | |
| Interpretation | 20 | Give a cautious overall interpretation of results considering objectives, limitations, multiplicity of analyses, results from similar studies, and other relevant evidence | | 15 | | | | (Line# 312-323)  Within the limitations of the study, FD analysis performed on the mandible demonstrated contradictory results on the trabecular and cortical regions. The basal cortical bone microarchitecture in a subpopulation of FMF patients revealed a decreased bone density. On the contrary, trabecular bone in the mandibular angle and condyle regions showed an increase in bone density. FMF disease might be a candidate for referral to DXA examination based on decreased bone density in the mandibular basal cortex detected by FD measurements on routine panoramic radiographs. According to our results, the relationship between colchicine use and the microarchitecture of mandibular bone is conjectural. Further studies performed on larger patient populations, in which the type of mutation resulted in FMF, the colchicine regimen and other factors affecting mandibular bone structure, such as parafunctional habits and chewing side preferences, were taken into consideration, are necessary to confirm these findings. | |
| Generalisability | 21 | Discuss the generalisability (external validity) of the study results | | 15 | | | | (Line# 316-323)  FMF disease might be a candidate for referral to DXA examination based on decreased bone density in the mandibular basal cortex detected by FD measurements on routine panoramic radiographs.  Further studies performed on larger patient populations, in which the type of mutation resulted in FMF, the colchicine regimen and other factors affecting mandibular bone structure, such as parafunctional habits and chewing side preferences, were taken into consideration, are necessary to confirm these findings. | |
| **Other information** | |  | | | | | | | |
| Funding | 22 | Give the source of funding and the role of the funders for the present study and, if applicable, for the original study on which the present article is based | | | | | - | | No funding was obtained neither for this study nor the original study on which the present article is based. |

*Give information separately for cases and controls in case-control studies and, if applicable, for exposed and unexposed groups in cohort and cross-sectional studies.

**Note:** An Explanation and Elaboration article discusses each checklist item and gives methodological background and published examples of transparent reporting. The STROBE checklist is best used in conjunction with this article (freely available on the Web sites of PLoS Medicine at http://www.plosmedicine.org/, Annals of Internal Medicine at http://www.annals.org/, and Epidemiology at http://www.epidem.com/). Information on the STROBE Initiative is available at www.strobe-statement.org.
