# Supplementary material for: Fractal dimension analysis of different mandibular regions in familial Mediterranean fever patients: A cross-sectional retrospective study
Source: PLoS One. 2023 Jun 30;18(6):e0288170. doi: 10.1371/journal.pone.0288170 (PMC10313079; doi:10.1371/journal.pone.0288170)
Supplement: S1 Protocol — (DOCX) [file pone.0288170.s002.docx]

**Fractal Dimension (FD) Analysis**

The fractal dimension (FD) analysis measurements were performed on the ImageJ software (ImageJ 1.38; US National Institutes of Health, Bethesda, MD, USA) that was downloaded from <https://imagej.nih.gov/ij/download.html>.

All sets of the fully anonymized panoramic images were imported into ImageJ software. Four different regions of interest (ROI) in 30x30 pixel size were selected from designated spots (rectangle tool) on right (R) and left (L) side of the mandible as follows [1] (Fig 1); ROI1: Distal region of the premolar, next to the mental foramen, ROI2: mesial region of the apical part of second molar, ROI3: angular region of the mandible, and ROI4: mandibular condyle area. An additional fifth ROI was selected in differing pixel size depending on the ROI (polygon tool) on each side of the mandible as; ROI5: basal cortical bone of the mandible extending from distal to the mental foramen to the distal root of the first molar.

**Fig 1. Designated region of interests (ROI) indicated on the panoramic radiograph.**


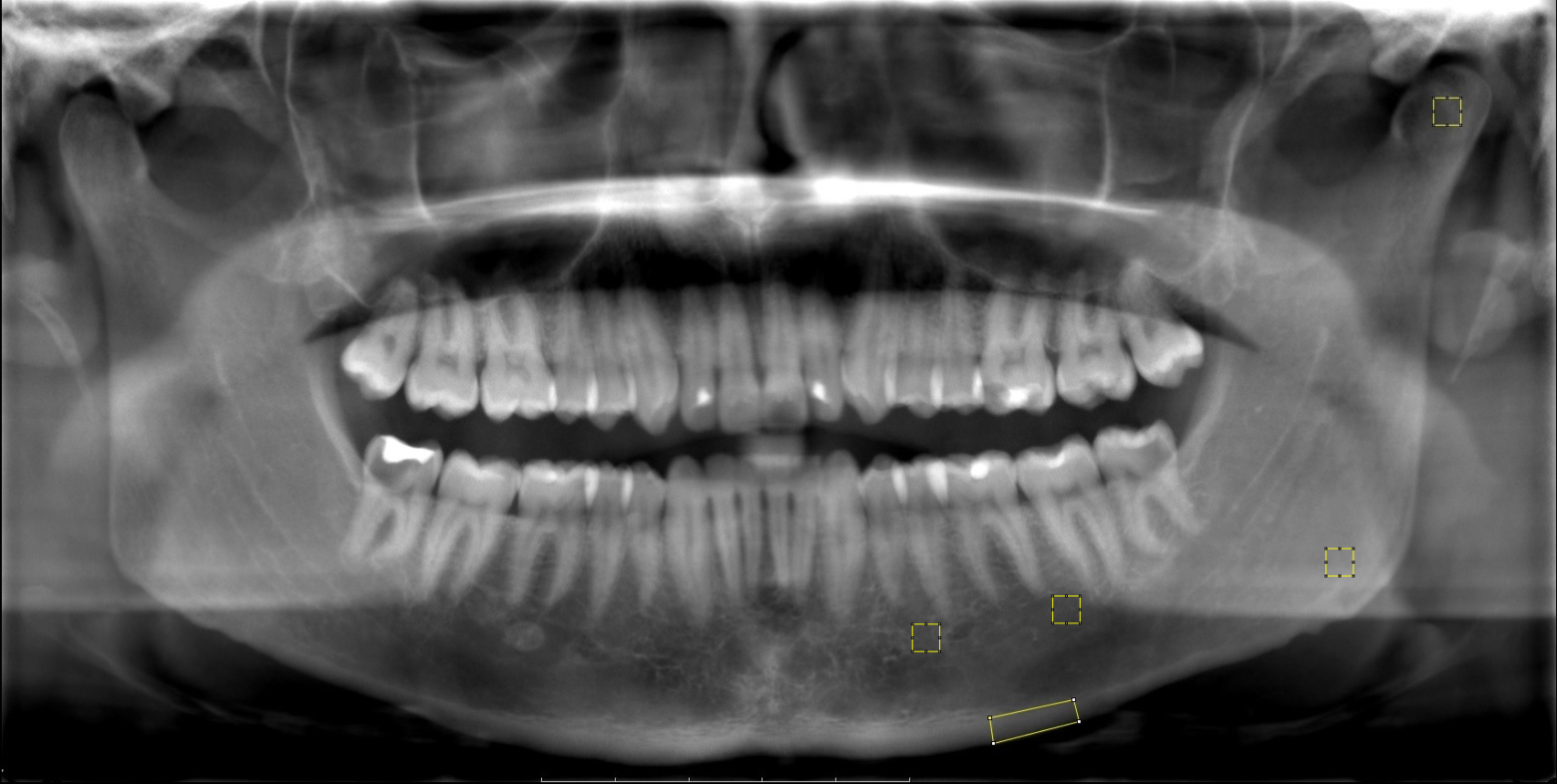


**ROI4**

**ROI1**

**ROI2**

**ROI3**

**ROI5**

Each ROI was selected and the FD analysis was conducted as follows; duplication of the selected ROI, application of the Gaussian filter to remove brightness alterations due to overlying soft and hard tissue, subtraction of the filtered image from the original cropped image, addition of a gray value of 128 to differentiate bone marrow spaces and trabeculae, binarization of the resulting image, steps of erosion, dilatation, inversion, and skeletonization. Lastly, the FD was calculated according to the fractal box counting method described by White & Rudolph [2].

**Briefly (Fig 2a-h)**;


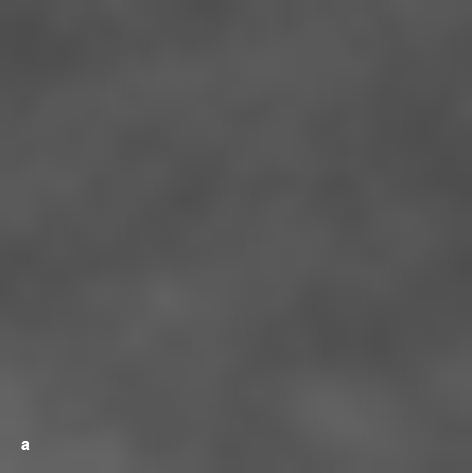


- Crop the selected region of interests (ROI)

and duplicate the ROI image.


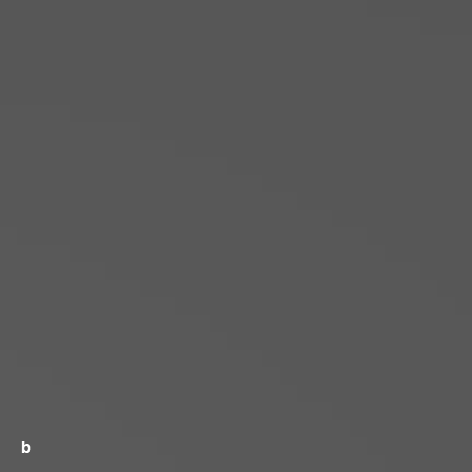


- Use Gaussan filter (sigma=35 pixels)

for blurred image


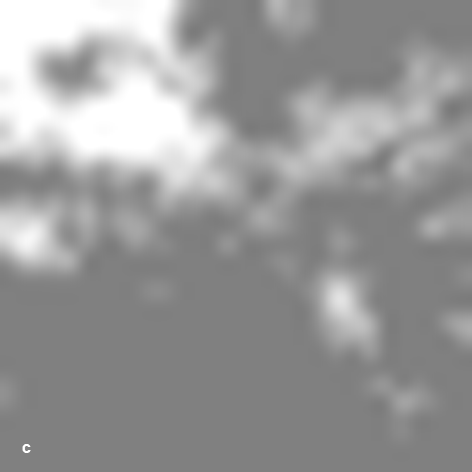


- Substract filtered image from original cropped image and change the image type to RGB color

- Add 128 grey value (Math 🡪 Tool 🡪 Add)


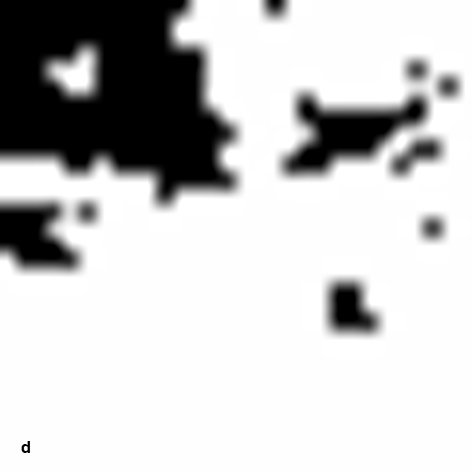


- Binary tool – make binary


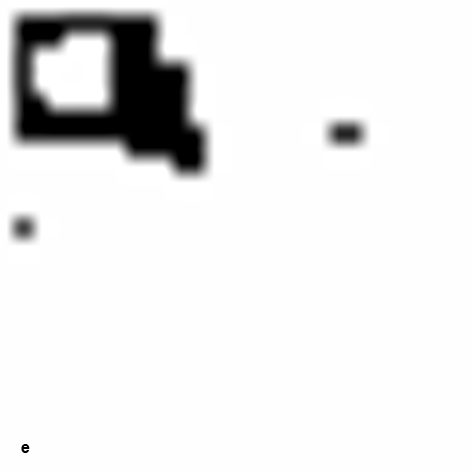


- Binary tool - Erode


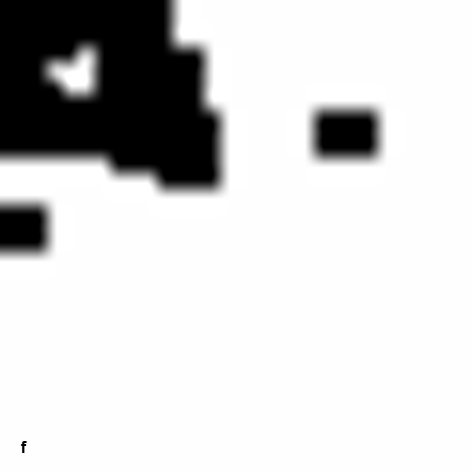


- Binary tool - Dilate


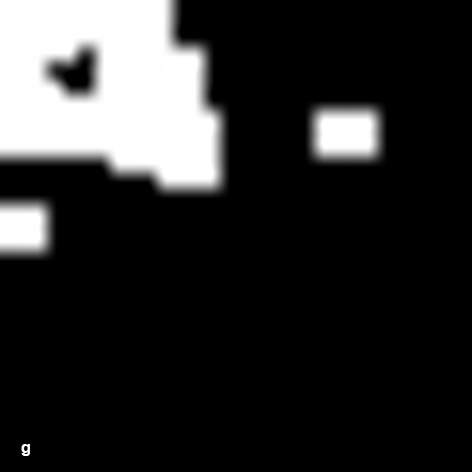


- Image inverter

(inverts black to white and vice versa)

- Binary tool 🡪 Skeletonize


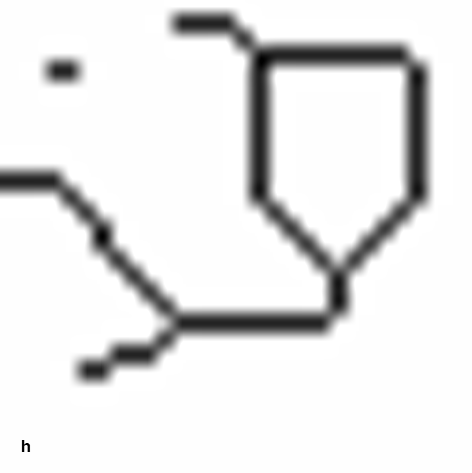


- Analyze 🡪Tools 🡪 Fractal box count

**References**

1. Yagmur B, Tercanli-Alkis H, Tayfun-Kupesiz F, Karayilmaz H, Kupesiz OA. Alterations of panoramic radiomorphometric indices in children and adolescents with beta-thalassemia major: A fractal analysis study. Med Oral Patol Oral Cir Bucal. 2022;27:e10-7.
2. White SC, Rudolph DJ. Alterations of the trabecular pattern of the jaws in patients with osteoporosis. Oral Surg Oral Med Oral Pathol Oral Radiol Endod. 1999;88:628-35.
