# Supplementary material for: Fractal dimension analysis of different mandibular regions in familial Mediterranean fever patients: A cross-sectional retrospective study
Source: PLoS One. 2023 Jun 30;18(6):e0288170. doi: 10.1371/journal.pone.0288170 (PMC10313079; doi:10.1371/journal.pone.0288170)
Supplement: Attachment — Submitted filename: Response to Reviewers.docx [file pone.0288170.s004.docx]

Jun 3, 2023

**Manuscript Reference No:** PONE-D-23-08206

**Title:** Prediction of decreased mandibular bone density in familial Mediterranean fever via fractal dimension analysis: A cross-sectional retrospective study

Dear Academic Editor and Reviewer(s),

Please find the responses to your comments and suggestions below. We are deeply appreciated that they assisted us to turn our manuscript to a far better version.

I have also made some additional corrections, such as grammatical or spelling errors and some other mistakes, that I have noticed during the revision of the manuscript. I would like to kindly ask you to consider them as a part of the revision process, as well.

Thank you for your time and considerations.

Kindest regards,

Dr. Nilüfer Ersan

Comments from the Academic Editor and Reviewer(s):

If applicable, we recommend that you deposit your laboratory protocols in protocols.io to enhance the reproducibility of your results. Protocols.io assigns your protocol its own identifier (DOI) so that it can be cited independently in the future. For instructions see: <https://journals.plos.org/plosone/s/submission-guidelines#loc-laboratory-protocols>. Additionally, PLOS ONE offers an option for publishing peer-reviewed Lab Protocol articles, which describe protocols hosted on protocols.io. Read more information on sharing protocols at <https://plos.org/protocols?utm_medium=editorial-email&utm_source=authorletters&utm_campaign=protocols>.

**We do not have a laboratory protocol. However, we have deposited our study protocol along with the other supporting materials in** [**https://doi.org/10.6084/m9.figshare.23259770**](https://doi.org/10.6084/m9.figshare.23259770).

1. Please ensure that your manuscript meets PLOS ONE's style requirements, including those for file naming. The PLOS ONE style templates can be found at https://journals.plos.org/plosone/s/file?id=wjVg/PLOSOne_formatting_sample_main_body.pdf and 
https://journals.plos.org/plosone/s/file?id=ba62/PLOSOne_formatting_sample_title_authors_affiliations.pdf

**Revised accordingly.**

2. Please note that PLOS ONE has specific guidelines on code sharing for submissions in which author-generated code underpins the findings in the manuscript. In these cases, all author-generated code must be made available without restrictions upon publication of the work. Please review our guidelines at https://journals.plos.org/plosone/s/materials-and-software-sharing#loc-sharing-code and ensure that your code is shared in a way that follows best practice and facilitates reproducibility and reuse.

**We have shared our supporting materials in** [**https://doi.org/10.6084/m9.figshare.23259770**](https://doi.org/10.6084/m9.figshare.23259770)**.**

3. We note that Figure (2) includes an image of a [patient / participant / in the study]. 
As per the PLOS ONE policy (http://journals.plos.org/plosone/s/submission-guidelines#loc-human-subjects-research) on papers that include identifying, or potentially identifying, information, the individual(s) or parent(s)/guardian(s) must be informed of the terms of the PLOS open-access (CC-BY) license and provide specific permission for publication of these details under the terms of this license. Please download the Consent Form for Publication in a PLOS Journal (http://journals.plos.org/plosone/s/file?id=8ce6/plos-consent-form-english.pdf). The signed consent form should not be submitted with the manuscript, but should be securely filed in the individual's case notes. Please amend the methods section and ethics statement of the manuscript to explicitly state that the patient/participant has provided consent for publication: “The individual in this manuscript has given written informed consent (as outlined in PLOS consent form) to publish these case details”.

If you are unable to obtain consent from the subject of the photograph, you will need to remove the figure and any other textual identifying information or case descriptions for this individual.

**We have successfully obtained consent from the individual and regarding this we inserted a statement in the Editorial Manager System and manuscript. We are keeping the file and did not submit it with the manuscript as instructed.**

[Note: HTML markup is below. Please do not edit.]

Reviewers' comments:

Reviewer's Responses to Questions

**Comments to the Author**

1. Is the manuscript technically sound, and do the data support the conclusions?

The manuscript must describe a technically sound piece of scientific research with data that supports the conclusions. Experiments must have been conducted rigorously, with appropriate controls, replication, and sample sizes. The conclusions must be drawn appropriately based on the data presented.

Reviewer #1: No

**There are no comments or suggestions to reply.**

2. Has the statistical analysis been performed appropriately and rigorously?

Reviewer #1: Yes

**There are no comments or suggestions to reply.**

3. Have the authors made all data underlying the findings in their manuscript fully available?

The [PLOS Data policy](http://www.plosone.org/static/policies.action#sharing) requires authors to make all data underlying the findings described in their manuscript fully available without restriction, with rare exception (please refer to the Data Availability Statement in the manuscript PDF file). The data should be provided as part of the manuscript or its supporting information, or deposited to a public repository. For example, in addition to summary statistics, the data points behind means, medians and variance measures should be available. If there are restrictions on publicly sharing data—e.g. participant privacy or use of data from a third party—those must be specified.

Reviewer #1: No

**We have shared our supporting materials in** [**https://doi.org/10.6084/m9.figshare.23259770**](https://doi.org/10.6084/m9.figshare.23259770)**.**

4. Is the manuscript presented in an intelligible fashion and written in standard English?

PLOS ONE does not copyedit accepted manuscripts, so the language in submitted articles must be clear, correct, and unambiguous. Any typographical or grammatical errors should be corrected at revision, so please note any specific errors here.

Reviewer #1: Yes

**There are no comments or suggestions to reply.**

5. Review Comments to the Author

Please use the space provided to explain your answers to the questions above. You may also include additional comments for the author, including concerns about dual publication, research ethics, or publication ethics. (Please upload your review as an attachment if it exceeds 20,000 characters)

Reviewer #1: I have read carefully the manuscript entitled “Prediction of decreased mandibular bone density in familial Mediterranean fever via fractal dimension analysis: A cross-sectional retrospective study.” However, Authors didn't draft the paper well and I cannot recommend the publication of the article in its present form. The Authors should correct the manuscript before the re-submission of the work to Plos One.

First of all, the title does not correspond to the content of the manuscript, as no "prediction" or "bone density" has been established. The study only focuses on the assessment of the FD (fractal dimension) in different regions of the mandibular bone.

**The title was revised accordingly as ‘Fractal dimension analysis of different mandibular regions in familial Mediterranean fever patients: A cross-sectional retrospective study’.**

Secondly, there is no paragraph in which it is clearly stated that the method used to determine FD can be a measure of bone BMD (L66 and others). As indicated, FD is commonly used to assess the homogeneity of trabecular bone microarchitecture, not only in the mandible, and it is more accurately a measure of the regularity of the surface of the trabeculae. Therefore, measuring FD in the cortical bone area no longer aligns with the correct definition of FD provided in L67 and L206-208. Thus, additionally give additional references about measurements of FD of cortical bone.

**The manuscript was redrafted emphasizing the use of FD in the BMD evaluation and in assessment of cortical bone.**

Minor comments:
L56 In which country (countries) ?

**Related information was inserted in the text.**

L102 n- 25 in each group

**The phrase ‘in each group’ was inserted.**

L132 “grey scale”

**The term ‘grayscale’ was inserted to the statement.**

L173 According to the data in the tables, all variables lack normal distribution. Am I right?

**In order to confirm the normality of the data, we have consulted to our statistician, who reevaluated the data in terms of different regions of interests and found that R3, R4, R5, L1, and L5 were normally distributed, while the others were not. As well, the data was reviewed and some minor corrections which did not affect the significance of the findings, were made. Abstract, Results section and related tables were revised accordingly. Thank you so much for your contribution.**

Also information about checking the groups with the Chi-square test is missing.

**Information regarding the Chi-square test was inserted to the Materials and methods section as: 'Chi-square test was used to assess the distribution of categorical data between FMF and control groups.’**

Tables - correct the titles of tables 3-5, because you did not measure ROI, only FD in specific areas of the mandible

**Titles of the Tables 3-6 were corrected.**

6. PLOS authors have the option to publish the peer review history of their article ([what does this mean?](https://journals.plos.org/plosone/s/editorial-and-peer-review-process#loc-peer-review-history)). If published, this will include your full peer review and any attached files.

**Yes.**

If you choose “no”, your identity will remain anonymous but your review may still be made public.


**Do you want your identity to be public for this peer review?** For information about this choice, including consent withdrawal, please see our [Privacy Policy](https://www.plos.org/privacy-policy).

Reviewer #1: No

 [NOTE: If reviewer comments were submitted as an attachment file, they will be attached to this email and accessible via the submission site. Please log into your account, locate the manuscript record, and check for the action link "View Attachments". If this link does not appear, there are no attachment files.]

**There were no additional attachment files to consider.**

While revising your submission, please upload your figure files to the Preflight Analysis and Conversion Engine (PACE) digital diagnostic tool, <https://pacev2.apexcovantage.com/>. PACE helps ensure that figures meet PLOS requirements. To use PACE, you must first register as a user. Registration is free. Then, login and navigate to the UPLOAD tab, where you will find detailed instructions on how to use the tool. If you encounter any issues or have any questions when using PACE, please email PLOS at [figures@plos.org](mailto:figures@plos.org). Please note that Supporting Information files do not need this step.

**I have arranged the figures in the PACE system. Nevertheless, I have sent an email to** [figures@plos.org](mailto:figures@plos.org) **and waiting for approval for the appropriateness of the figures.**

Thank you for your time and consideration.
